# HIF-1alpha and HIF-2alpha Are Differentially Activated in Distinct Cell Populations in Retinal Ischaemia

**DOI:** 10.1371/journal.pone.0011103

**Published:** 2010-06-14

**Authors:** Freya M. Mowat, Ulrich F. O. Luhmann, Alexander J. Smith, Clemens Lange, Yanai Duran, Sarah Harten, Deepa Shukla, Patrick H. Maxwell, Robin R. Ali, James W. B. Bainbridge

**Affiliations:** 1 Department of Genetics, University College London Institute of Ophthalmology, London, United Kingdom; 2 Division of Medicine, University College London, London, United Kingdom; University of Oldenburg, Germany

## Abstract

**Background:**

Hypoxia plays a key role in ischaemic and neovascular disorders of the retina. Cellular responses to oxygen are mediated by hypoxia-inducible transcription factors (HIFs) that are stabilised in hypoxia and induce the expression of a diverse range of genes. The purpose of this study was to define the cellular specificities of HIF-1alpha and HIF-2alpha in retinal ischaemia, and to determine their correlation with the pattern of retinal hypoxia and the expression profiles of induced molecular mediators.

**Methodology/Principal Findings:**

We investigated the tissue distribution of retinal hypoxia during oxygen-induced retinopathy (OIR) in mice using the bio-reductive drug pimonidazole. We measured the levels of HIF-1alpha and HIF-2alpha proteins by Western blotting and determined their cellular distribution by immunohistochemistry during the development of OIR. We measured the temporal expression profiles of two downstream mediators, vascular endothelial growth factor (VEGF) and erythropoietin (Epo) by ELISA. Pimonidazole labelling was evident specifically in the inner retina. Labelling peaked at 2 hours after the onset of hypoxia and gradually declined thereafter. Marked binding to Müller glia was evident during the early hypoxic stages of OIR. Both HIF-1alpha and HIF-2alpha protein levels were significantly increased during retinal hypoxia but were evident in distinct cellular distributions; HIF-1alpha stabilisation was evident in neuronal cells throughout the inner retinal layers whereas HIF-2alpha was restricted to Müller glia and astrocytes. Hypoxia and HIF-alpha stabilisation in the retina were closely followed by upregulated expression of the downstream mediators VEGF and EPO.

**Conclusions/Significance:**

Both HIF-1alpha and HIF-2alpha are activated in close correlation with retinal hypoxia but have contrasting cell specificities, consistent with differential roles in retinal ischaemia. Our findings suggest that HIF-2alpha activation plays a key role in regulating the response of Müller glia to hypoxia.

## Introduction

Ischaemia is common to the major causes of blindness including diabetes and retinopathy of prematurity. Ischaemia induces powerful endogenous responses to protect against tissue injury, including compensatory changes in blood flow, paracrine expression of neurotrophic factors and angiogenesis. In the eye, however, angiogenesis can be disorganised and typically results in oedema and haemorrhage that adversely affect visual function. There is an unmet need for therapies that promote endogenous protective responses and prevent harmful angiogenesis. The development of such strategies depends on a clear understanding of oxygen sensing mechanisms in the retina and the roles of downstream mediators.

The principal regulator of the transcriptional response to hypoxia is the hypoxia-inducible factor (HIF) family of transcription factors [1], [2]. HIF is a heterodimeric transcription factor, composed of one of the 3 oxygen-sensitive HIF-alpha subunits (HIF-1alpha, HIF-2alpha and HIF-3alpha) and the oxygen-insensitive and constitutively expressed HIF-beta subunit (ARNT) In normoxic conditions hydroxylated HIF-alpha is bound to von Hippel-Lindau protein (pVHL) and is targeted for ubiquitination and subsequent proteosomal degradation [3]–[5]. Under hypoxic conditions, dimerisation of the stabilised HIF-alpha subunit with the HIF-beta subunit enables the transcriptional activity of a wide range of genes including those involved in cellular metabolism, hypoxia tolerance and angiogenesis, such as vascular endothelial growth factor (VEGF) and erythropoietin (EPO) [6]–[8]. Although the stabilisation of HIF-1alpha in hypoxia is largely controlled by the inhibition of the VHL degradation pathway, an increase in translation by stabilisation of *HIF* mRNA also occurs in certain cell types [9]–[11]. The role of HIF-3alpha is yet to be clearly defined, but may involve the adaptive response to hypoxia through regulation of other HIF isoforms [12].

While HIF-1alpha and HIF-2alpha subunits are highly homologous and structurally similar in their DNA binding and dimerisation domains, they have distinct roles both during development [13]–[15] and in adaptive responses to hypoxia [8], [11], [16], [17]. Their dissimilar roles may reflect differences in cellular distribution[11], [16], transcriptional regulation [18], [19] and co-activation or repression [20]. Evidence from *in vitro* studies suggests that HIF-1alpha responds only to severe hypoxia whereas HIF-2alpha is stabilised in relatively moderate hypoxia [11]. The cellular distributions of HIF-alpha isoforms within the hypoxic retina, and their relative timecourses of stabilisation are not clearly defined. The purpose of the present study was to compare the cellular specificities of HIF-1alpha and HIF-2alpha in retinal ischaemia, and to determine their spatiotemporal correlation with retinal hypoxia and the expression profiles of induced molecular mediators. The results demonstrate that both HIF-1alpha and HIF-2alpha are upregulated by post-translational stabilization in close correlation with hypoxia but have dissimilar cellular specificities. The distinct spatial distributions of HIFs provide further evidence that HIF-1alpha and HIF-2alpha have isoform-specific roles in cellular adaptation to hypoxia in the retina.

## Results and Discussion

### The spatial and temporal pattern of hypoxia in OIR

To investigate the effect of hypoxia on the HIF pathway in the retina we used a well-characterised mouse model of oxygen-induced retinopathy (OIR) [21]. Briefly, mice exposed to 75% oxygen from postnatal day 7 (p7) to p12 develop areas of retinal capillary obliteration; on return to room air at p12 the ischaemic retina becomes hypoxic until it is revascularised at around p26. To determine the tissue distribution and timecourse of hypoxia in relation to vascularisation, we co-stained flatmounted retinas of animals at intervals during OIR development with the hypoxia marker pimonidazole and the vascular endothelial marker *BS* lectin. Previous studies in animal models have demonstrated that oxygen tensions can fall below 5 mmHg in the ischemic retina [22]–[24], a level well within the detection range of pimonidazole that forms an adduct with hypoxic proteins at a pO_2_ of less than 10 mmHg [25]–[28].

**Figure 1:**
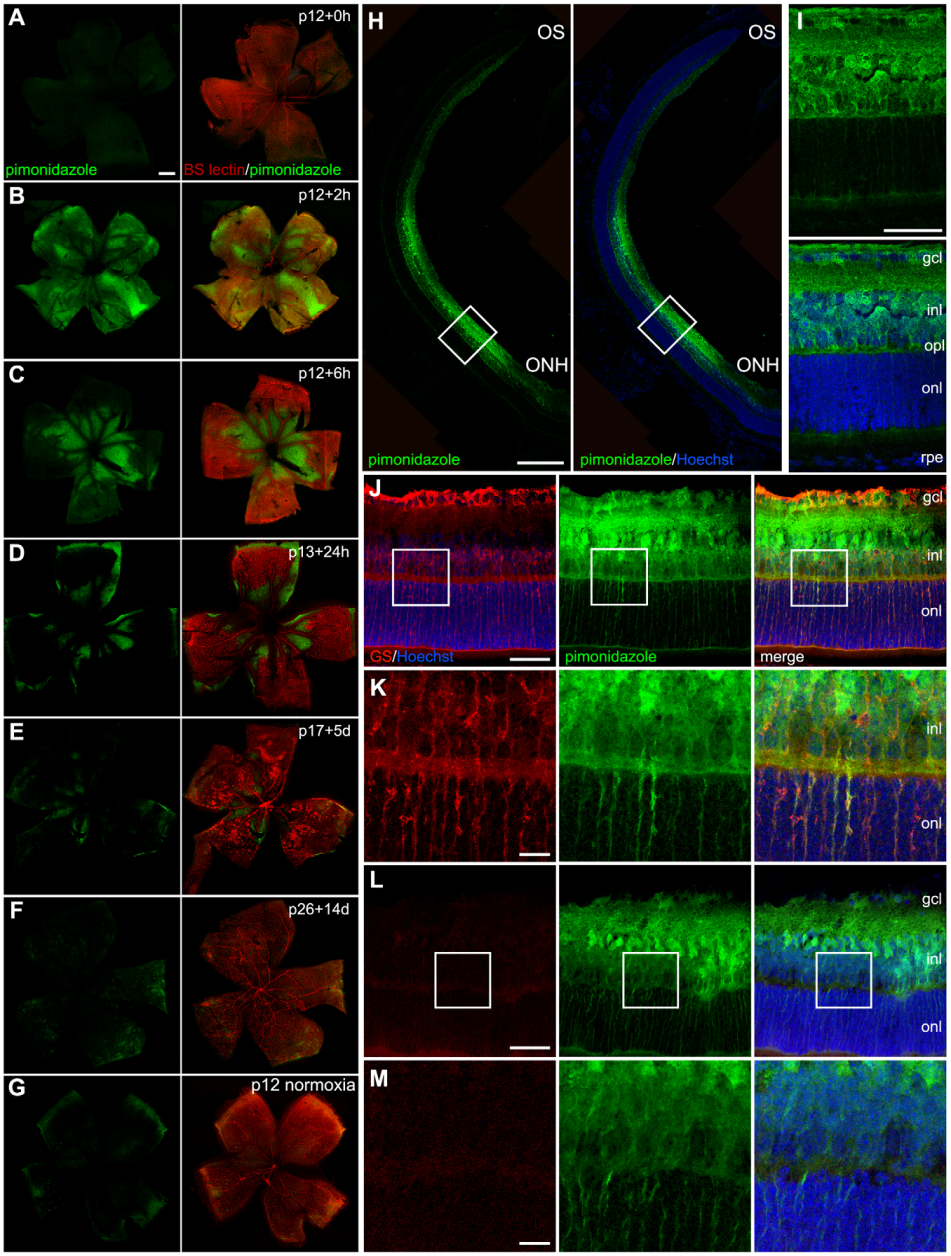
Temporal and spatial distribution of hypoxia throughout oxygen-induced retinopathy (OIR) development. Representative retinal flatmounts throughout OIR development are shown in A–G (magnification ×5) co-stained for hypoxia (pimonidazole, green) and vasculature (*Bandeiriae simplicifolia* lectin, red); A, 0 h; B, 2 h; C, 6 h; D, 24 h (p13); E, 5 days (p17); F, 14 days (p26); G, normoxic control. Representative retinal cryosections at the p12+2 h timepoint are shown in detail in H–M. Marked pimonidazole staining (green) was evident in the inner retina, and was strongest in the central region corresponding with ischaemia (H, magnification ×20; I, magnification ×63). Strong pimonidazole staining was evident in all tissue layers of the inner retina including the outer plexiform layer. Pimonidazole staining apparent in a linear radial configuration in the outer retina co-localised closely with the Müller glial marker glutamine synhetase (red) (x40 magnification J, enlarged in K). A section incubated without glutamine synthetase primary antibody is shown in L (magnification ×40), enlarged in M. and minimal staining was detected in the outer nuclear layer and retinal pigment epithelium. ONH  =  optic nerve head, OS  =  ora serrata, gcl  =  ganglion cell layer, inl  =  inner nuclear layer, opl  =  outer plexiform layer, onl  =  outer nuclear layer, rpe  =  retinal pigment epithelium. Scale bars: in A (for A–G)  = 500 µm, H = 250 µm, I, J, L = 50 µm, K, M = 10 µm.

In animals at p12 still in 75% oxygen, the absence of pimonidazole labelling indicated no areas of retinal hypoxia (Figure 1A). In animals 2 hours following return to room air, strong pimonidazole labelling was evident throughout the retina indicated hypoxia of less than 10 mmHg. Hypoxia was evident not only within the ischaemic regions but also, to a lesser extent, in areas of persistent vascularisation. (Figure 1B). Six to 24 hours following return to room air, pimonidazole labelling was limited specifically to areas of capillary ablation in the central retina (Figure 1C and D). At p17 small areas of pimonidazole staining indicated hypoxia in persistently ischemic areas (Figure 1E). At p26 the retina was fully revascularised and there was no evidence of persistent hypoxia (Figure 1F). In animals at p12 that had been raised in room air throughout, pimonidazole staining evident only in the far retinal periphery indicated the physiologically low oxygen tension that is associated with normal retinal vascular development (Figure 1G).

The extent of hypoxia detection in the retina following OIR is consistent with results of other studies of murine and rat OIR [26]–[30] and further defines the temporal and spatial pattern of hypoxia in relation to vascular ablation and recovery in this model. In addition, we observed that during the first few hours after return to room air, retinal hypoxia extends well beyond the areas of capillary loss. The subsequent restriction of pimonidazole staining specifically to area of ischaemia suggests that hypoxia within vascularised regions is rapidly redressed by compensatory mechanisms, and we speculate that this may involve autoregulation of the inner retinal vascular supply.

To examine the distribution of hypoxia across the retinal layers we stained retinal sections from animals 2 hours following return to room air with pimonidazole. Staining was localised primarily to the inner retina, extending from the inner retinal surface to the outer border of the outer plexiform layer (Figure 1H and I). These findings are consistent with the considerable reduction in inner retinal oxygenation measured using micro-electrodes within the normal retina [31], and the pattern of hypoxia is consistent with measurements of retinal oxygenation during occlusions of the inner retinal circulation [32]. The relative specificity of pimonidazole labelling of the inner retina is consistent with insufficiency of the retinal circulation with sparing of the choroidal circulation that can support highly metabolically active photoreceptors but is insufficient to meet the oxygen demands of the inner retina [27]. At high magnification, pimonidazole staining in the outer retina was evident as linear processes extending through the outer nuclear layer and terminating at the outer limiting membrane. Co-localisation with the Müller cell marker glutamine synthetase indicated that these are processes of Müller glia. (Figure 1J; magnified in K) The presence of pimonidazole positive Müller cell processes in the outer nuclear layer may reflect intracellular movement of hypoxic adducts from the inner retina during the 3-hour period between pimonidazole administration and analysis.

### The spatial and temporal distribution of HIF-1alpha and HIF-2alpha in OIR

**Figure 2:**
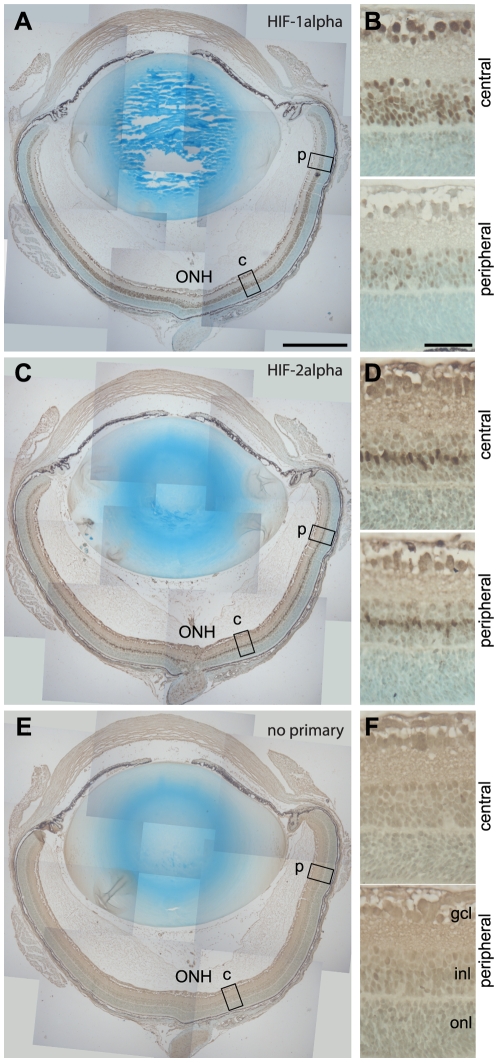
Spatial distribution of HIF-1alpha and HIF-2alpha in the early hypoxic phase of OIR. Immunohistochemistry for HIF-1alpha and HIF-2alpha was performed on 6 µm paraffin sections of eyes from animals that had undergone OIR, culled 2 hours after removal from hyperoxia (p12+2 h, n = 6 independent eyes). The secondary antibody was detected by DAB staining. Masson's light green was used as a connective tissue counterstain. HIF-1alpha was detected throughout the inner retina, particularly in the central central region corresponding to the area of greatest hypoxia (x20 magnification shown in A, and ×40-magnified images shown in B). The spatial distribution of HIF-2alpha on an adjacent retinal section was very similar but restricted to a small number of cells of the inner retina (x20 magnification shown in C, ×40 magnified images shown in D). A serial section incubated without primary antibody showed no specific staining (Figure 2E and F). ONH  =  optic nerve head, gcl  =  ganglion cell layer, inl – inner nuclear layer, onl  =  outer nuclear layer. Scale bars: in A (for A, C, E): 500 µm, in B (for B, D, F): 50 µm.

To compare the tissue distribution of HIF-1alpha and HIF-2alpha with that of hypoxia we performed immunohistochemistry on retinal sections in mice 2 hours following return to room air. At this timepoint, immunostaining for both HIF-1alpha (Figure 2A and B) and HIF-2alpha (Figure 2C and D) was evident in the inner retina, and was more prominent in the central retina than in the periphery. The tissue distributions are consistent with the stabilisation of HIFs in areas of retinal hypoxia indicated by pimonidazole labelling in retinal flatmounts.

**Figure 3:**
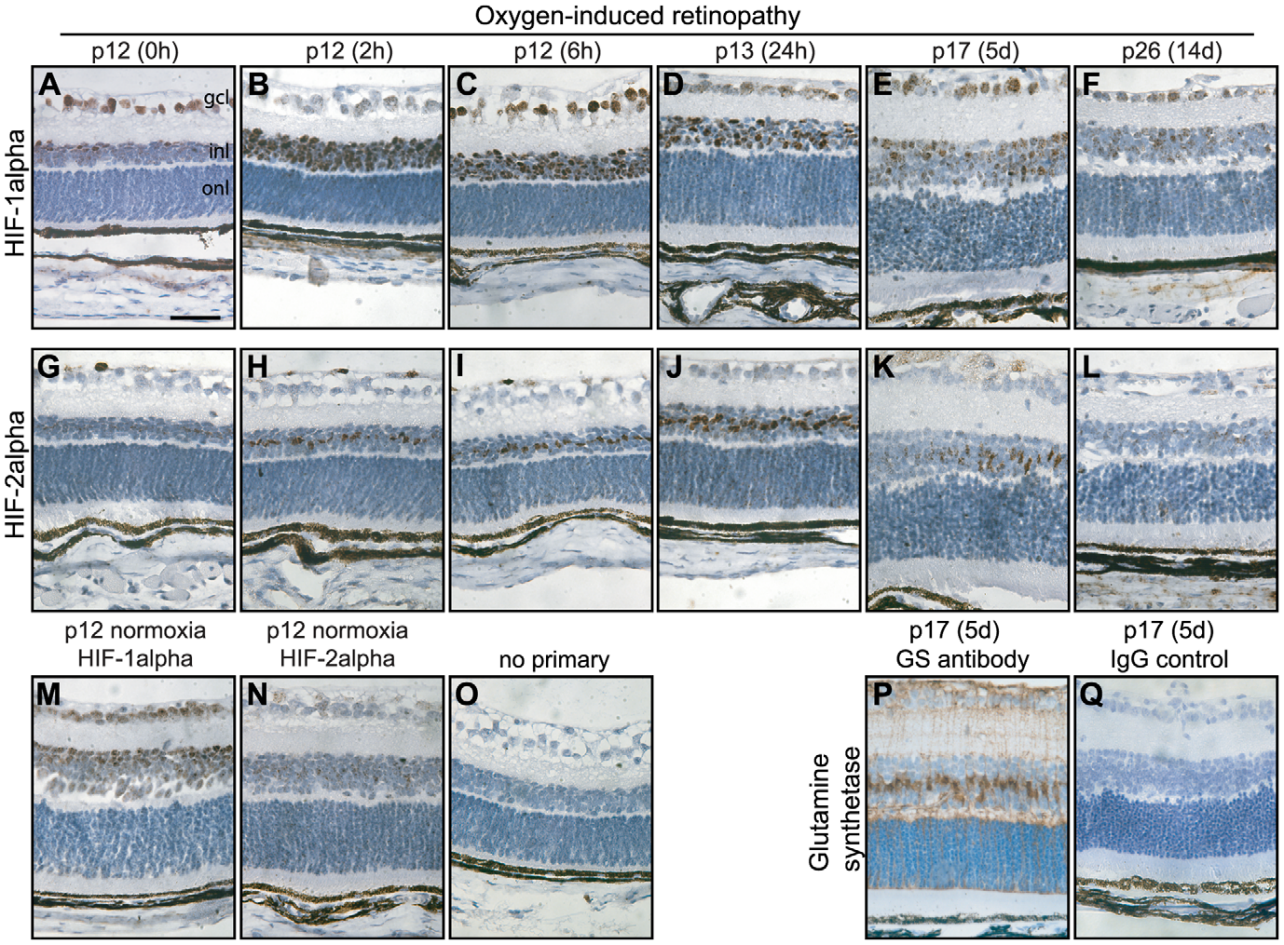
Temporal distribution of HIF-1alpha and HIF-2alpha throughout OIR development. Immunohistochemistry for HIF-1alpha (A–F) and HIF-2alpha (G–L) was performed throughout OIR development, using DAB staining and haematoxylin counterstain. The central region (2 high power fields from the optic nerve head) from central retinal sections at ×40 magnification is shown. Normoxic controls at p12 are shown for HIF-1alpha (M) and HIF-2alpha (N). A section at p12+2 h is shown where the primary antibody was omitted is shown in O; the laminar dark brown appearance in the outer retina reflects pigment in the retinal pigment epithelium and choroid. Immunohistochemistry for the Müller cell marker glutamine synthetase performed on a serial section at p17 (P) strongly suggests that the upregulation of HIF-2alpha in this model is highly restricted to Müller glia. Figure 3Q shows a serial section to P, incubated with a non-targeting IgG control primary antibody raised in the same species as the anti-glutamine synthetase antibody. Gcl  =  ganglion cell layer, inl  =  inner nuclear layer, onl  =  outer nuclear layer. Scale bar in A: 50 µm.

To investigate the detailed cellular specificities and timecourses of HIF-1alpha and HIF-2alpha in retinal ischaemia we examined sections of central retina from mice at intervals following return from hyperoxia to room air. In animals at p12 still in 75% oxygen, immunostaining for both HIF-1alpha and HIF-2alpha was detected at low levels in the inner retina (Figure 3A and G) comparable to staining in mice raised in room air throughout (Figure 3 M, N). Two hours following return to room air, HIF-1alpha and HIF-2alpha proteins were strongly evident in nuclei of cells in the inner retina (Figure 3B and H). Similar staining was observed at 6 and 24 hours (HIF-1alpha: Figure 3C and D, HIF-2alpha: Figure 3I and J). After 5 days following return to room air the degree of HIF-alpha staining was considerably reduced (Figure 3E and K), and after 2 weeks was comparable to baseline (Figure 3F and L compared with 3A and 3G).

Stabilisation of both HIF-1alpha and HIF-2alpha was restricted to the inner retina. Evidence of HIF-1alpha and HIF-2alpha in the outer retina was weak throughout OIR, consistent with the provision of oxygen to photoreceptor cells by the uncompromised choroidal circulation. This pattern of HIF regulation is in contrast to the effect of systemic hypoxia in which upregulation of HIF-1alpha extends across the outer retina [33].

Strikingly, HIF-1alpha and HIF-2alpha were evident in contrasting cellular distributions within the inner retina. HIF-1alpha staining was prominent in cells across the inner nuclear layer and in retinal ganglion cells. In contrast, HIF-2alpha was highly restricted to a discrete layer of cells within the inner nuclear layer, and to occasional astrocytes in the nerve fibre layer. Immunostaining of an adjacent section for the Müller cell marker glutamine synthetase at p17 demonstrated a very similar distribution of cells (Figure 3P), strongly suggesting that upregulation of HIF-2alpha in this model is specific to Müller glia.

### The temporal correlation of *HIF-1alpha* and *HIF-2alpha* RNA and protein in OIR with the upregulated expression of downstream mediators VEGF and Epo

**Figure 4:**
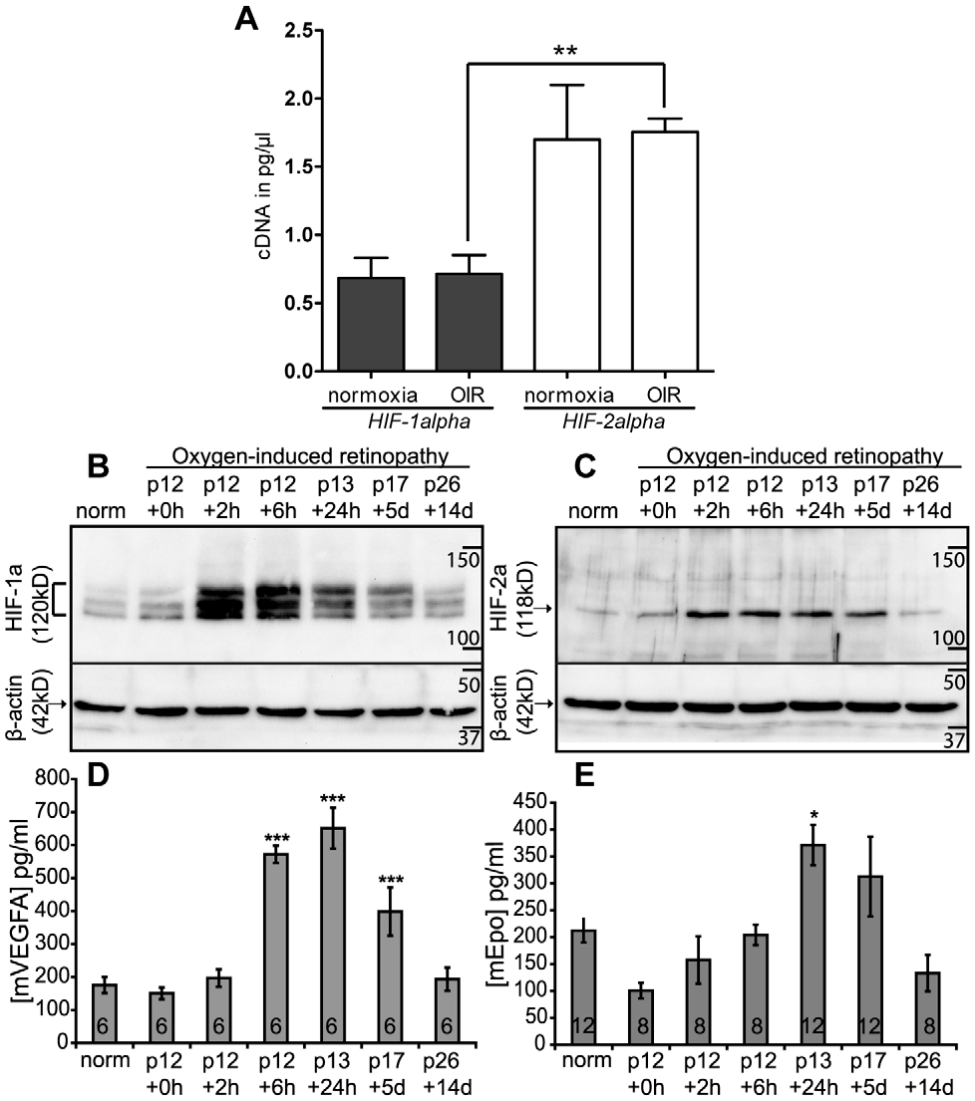
HIF-alpha expression and protein stabilisation in OIR and relation to ocular VEGF and Epo expression. Retinal RNA was isolated from eyes taken at p13 from animals which had undergone OIR (n = 3) and age-matched normoxic controls (n = 3). RT-PCR standard curves were generated and Ct values of normoxic and hypoxic retinal cDNA were compared to the standard curves (A). Western blots for HIF-1alpha (B) and HIF-2alpha (C) on retinal protein extracts showed that both proteins were strongly upregulated within 2 h of hypoxia. HIF-1alpha was detected as multiple bands, whereas HIF-2alpha was detected as a single band. HIF-alpha upregulation was maintained at p17 but not by p26. mVEGFA, measured by ELISA (D), was significantly upregulated within 6 hours of return to room air, reaching a peak at 24 hours. mEpo, (E), was also maximally upregulated at 24 hours although the induction of mEpo was less profound and more variable than mVEGFA. Levels of both proteins returned to normoxic levels by p26. ELISA values are expressed as concentration (pg/ml) per milligram of whole eye protein. * p<0.05, **p<0.01, *** p<0.001 unpaired t test in A, one-way ANOVA compared with p12 normoxia in D, E. p  =  postnatal day + time after removal from hyperoxia, norm  =  normoxia p12. In D and E, the number of eyes analysed at each time-point is noted within the bar in each graph. Graphs showmean ± SEM.

To investigate the expression of *HIF-1alpha* and *HIF-2alpha* in OIR we performed real-time RT-PCR on retinal cDNA from animals at p13. Total retinal *HIF-1alpha* was significantly greater than *HIF-2alpha* in both normoxia and OIR (Figure 4A). OIR was associated with no significant effect on the amount of either *HIF-1alpha* or *HIF-2alpha* RNA detected in the retina (Figure 4A) but caused a dramatic increase in the amount of HIF-1alpha and HIF-2alpha protein (Figure 4B–E) that correlated closely with the time course of protein upregulation demonstrated by immunohistochemistry.

Evaluation of HIF-alpha proteins by Western blotting suggested a subtle increase immediately on removal from hyperoxia (4B and 4C; p12+0 h). This most likely reflects the rapid response of the HIF system to the brief period of relative hypoxia in room air prior to tissue processing. HIF-alpha is stabilised within 1–2 hours of hypoxia *in vivo* [16], [17] and can accumulate within minutes [34], [35]. Alternatively, the subtle increase in HIF-alpha proteins at this time point may reflect hypoxia-independent mechanisms of HIF stabilisation by reactive oxygen species [36] or cytokines [8].

The lack of a detectable effect of OIR on RNA levels of either HIF-alpha isoform demonstrates that the upregulation and differential distribution of HIF-1alpha and HIF-2alpha transcription factors in retinal ischaemia result primarily from cell type and protein specific post-translational stabilisation, and not from differentially upregulated expression. This is consistent with the mechanisms described in acute hypoxia in the retina [35] and in other tissues, [17], [37] though increased stabilisation of mRNA may be involved in the retinal response to sustained systemic hypoxia [38].

To investigate the consequences of HIF-1alpha and HIF-2alpha activation in the retina we investigated the expression time-courses of their downstream mediators VEGF and Epo. The levels of both VEGF and Epo proteins in the whole eye were rapidly and significantly upregulated from p12 to p17. The profiles of protein upregulation were closely correlated to the timing of both HIF-1alpha and HIF-2alpha stabilisation in the retina (Figure 4F, G) and suggest no clear difference in transcriptional targeting.

HIF-1alpha is ubiquitously expressed. We found that HIF-1alpha is stabilised throughout the hypoxic inner retina, in ganglion cells and those of the inner nuclear layer, consistent with the distribution of HIF-1alpha described in previous reports [39], [40]. HIF-1alpha stabilisation in neuronal cells has been shown to be important for cell survival in the hypoxic brain [41] and retina [42], [43]. Therefore, in retinal ischaemia, HIF-1alpha stabilisation may have an important role in neuroprotection. HIF-2alpha was originally identified in vascular endothelial cells but is also expressed in a range of cells including interstitial cells and astrocytes [37], [44]. In the mouse retina, the expression of a surrogate marker suggests that HIF-2alpha is expressed by cells in the ganglion cell layer, inner nuclear layer and retinal pigment epithelium [45]. We have found that *HIF-2alpha* is expressed at a higher level than *HIF-1alpha* in the retina, and during retinal ischaemia is specifically activated in restricted populations of cells within the area of inner retinal hypoxia, consistent with Müller glia in the inner nuclear layer and astrocytes in the nerve fibre layer. Interestingly, the expression of both VEGF mRNA and Epo during ischaemia in OIR have a strikingly similar non-uniform distribution, with expression highly localized to cells in the centre of the inner nuclear layer and in the nerve fibre layer [39], [40], [46]. Recent data demonstrate that Müller cell derived VEGF is a significant contributor to retinal neovascularisation in OIR [47]. Our findings suggest that the response of Müller cells to hypoxia is mediated by activation of HIF-2alpha and provide the basis for future mechanistic and interventional studies.

HIF-2alpha mediates powerful angiogenic and neuroprotective responses to hypoxia. Haploinsufficiency of HIF-2alpha results in blunted induction of both VEGF and Epo, and reduced retinal neovascularisation in OIR [48]. HIF-2 is specifically responsible for the hypoxic upregulation of Epo [40], [49], [50] that may promote astrocytic paracrine-dependent neuronal survival during ischaemia [44], [51]–[54]. In the retina, HIF-2alpha induced expression of Epo may be responsible for the neuroprotective effect of hypoxic preconditioning [33]. HIF-2alpha has also been implicated mitochondrial homeostasis and regulation of antioxidant enzymes [55]. In the retina, deficiency of HIF-2alpha results in a retinal phenotype similar to oxidative-stress induced mitochondrial disease. [45]

This study demonstrates that both HIF-1alpha and HIF-2alpha are activated in close correlation with retinal hypoxia but have contrasting cell specificities within the inner retina. The findings suggest that HIF-1alpha and HIF-2alpha have differential roles in retinal ischaemia, and specifically that HIF-2alpha activation plays a key role in regulating the response of Müller glia to hypoxia.

## Methods

### Animals

All animals were used with University College London ethics committee approval and under a UK Home Office project licence and personal licence. All procedures were performed in accordance with the Association for Research in Vision and Ophthalmology Statement for the Use of Animals in Ophthalmic and Vision Research.

### The mouse model of oxygen-induced retinopathy (OIR)

Nursing mothers and their pups were placed in a 75% oxygen supply chamber from postnatal day 7 to postnatal day 12 as previously described [21]. A constant low flow of 80% oxygen, balance nitrogen was provided to a closed perspex chamber. The oxygen level was monitored, and maintained at 75% +/− 3% O_2_. Mice were checked twice daily, the mothers were removed from the chamber to breathe room air (21% oxygen) for a minimum of 2 hours a day to minimise lung toxicity associated with hyperoxia in adults. Food, water and bedding were changed every 2 days. The mice were exposed to a standard 12 hour light-dark cycle, and all euthanasia was performed in the light.

### Immunohistochemistry for pimonidazole (Hypoxyprobe ™)

60 mg/kg bodyweight pimonidazole hydrochloride (Hypoxyprobe-1™ kit, Chemicon (Millipore), Livingston UK) diluted in sterile phosphate buffered saline (PBS, Invitrogen Ltd. Paisley UK) was administered by intraperitoneal injection 3 hours prior to euthanasia as previously described [26]. Animals were terminally anaesthetised and eyes were fixed by intracardiac perfusion with 1% paraformaldehyde in PBS pH 7.4 and post fixed in 1-4% paraformaldehyde at room temperature. Retinal cryosections were incubated for 1 hour at room temperature in PBS with 1% bovine serum albumin (BSA, Sigma Aldrich Ltd. Gillingham, UK) and 5% normal goat serum (Abd serotec, Kidlington UK) and for retinal flatmounts, 0.05% (v/v) Triton X-100 (Sigma Aldrich Ltd., Gillingham UK) was added to the blocking solution. For retinal cryosection analysis (n = 3 eyes for each time-point), 12 µm retinal sections were incubated with anti-pimonidazole antibody (FITC conjugated mouse anti pimonidazole in Hypoxyprobe-1™ kit, Chemicon, Millipore Ltd. Livingston UK) at 1∶50 dilution in blocking solution for 1 hour at room temperature. For retinal flatmount examination (n = 3 eyes for each time-point), eyecups with the cornea and lens removed were incubated with anti-pimonidazole antibody at a dilution of 1∶100 in blocking solution for 4 hours at 37°C. Counterstaining with TRITC conjugated *Bandeiraea simplicifolia* (*BS*) lectin (Sigma Aldrich, Gillingham, UK) was performed at a concentration of 0.1 mg/ml in PBS overnight at 4°C. Retinas were washed extensively in PBS and dissected from the eyecup, radial cuts were made to flatten the retina, and the retinas were mounted with fluorescent aqueous mounting medium (Dako Ltd., Ely UK) ganglion cell layer uppermost using a coverslip.

### HIF-1alpha and HIF-2alpha immunohistochemistry

Following terminal anaesthesia (pentobarbital, Euthatal™ Merial Animal Health Ltd. Harlow, UK) eyes were fixed by intracardiac perfusion with ice-cold neutral buffered 10% (v/v) formalin. Post fixation was performed for 24 hours using formalin at 4°C, followed by 100% ethanol for 24 hours at 4°C. Eyes (n = 3 eyes for each time-point), were processed in an automated machine (Leica TP1020) then embedded in paraffin blocks for sectioning. Sections were cut on a microtome at 6 microns, and mounted on polylysine coated slides (Thermofisher Loughborough, UK). Antigen retrieval was performed using a commercial target retrieval solution (Dako Ltd., Ely UK) at 120°C. Immunohistochemistry was performed using a commercial signal amplification kit (Dako Ltd., Ely UK) according to the manufacturer's instructions, with diamino benzidine (DAB) as the final substrate. Primary antibodies and dilutions used are shown in Table 1. Primary antibodies were diluted in commercial antibody diluent (Dako Ltd., Ely UK) and were incubated at room temperature for 1 h as part of the signal amplification protocol. Sections were counterstained with either Masson's light green (staining connective tissue) or haematoxylin (staining nuclei), and mounted using DPX (VWR, Leicester UK).

**Table 1:** HIF-1alpha and HIF-2alpha primary antibodies used for immunohistochemistry and western blotting.

Antigen	Species and source of antibody	Use	Dilution
HIF-1alpha	Polyclonal rabbit anti-HIF-1alpha(Novus biologicals, Littleton USA) [16]	Immuno-histochemistry and western blotting	1∶10 000 (IHC)1∶ 500 (WB)
HIF-2alpha	PM8 rabbit antiserum [37]	Immuno-histochemistry	1∶20 000
HIF-2alpha	PM9 rabbit antiserum [37]	Western blotting	1∶2000
*β*-actin	Monoclonal mouse anti-β-actin (Sigma Aldrich, Gillingham UK)	Western blotting	1∶5000

### Other paraffin immunohistochemistry

Antibody staining for the Müller glial cell marker Glutamine Synthetase (GS) was performed at a concentration of 1∶100 after antigen retrieval as detailed above (mouse MAb anti-Glutamine Synthetase, BD Transduction Laboratories, Oxford UK). Blocking and primary antibody incubation was performed in 4% (v/v) NGS 1% (w/v) BSA PBS tween 0.05% (Sigma Aldrich, Gillingham UK) for 1 hour at room temperature. The primary antibody was biotinylated (Animal Research Kit, Dako UK Ltd.) and DAB was used as the final substrate after streptavadin-horseradish peroxidase incubation according to the manufacturer's recommendations.

### Real-time RT PCR

RNA was extracted from mouse tissue (n = 3 eyes for each group) using a commercial kit (RNeasy mini kit, Qiagen Ltd. Crawley UK). Tissue for RNA extraction was placed directly in lysis solution for immediate RNA purification, or placed in RNA stabilisation solution (RNAlater, Ambion, Warrington UK) and stored at 4°C for up to 2 weeks until RNA purification. Purified RNA was stored in DEPC treated water (Invitrogen Ltd. Paisley UK) at −80°C until use. The amount of template RNA was measured using a commercial small-volume spectrophotometer (Nanodrop ND-1000, ThermoScientific, Wilmington, DE USA). Up to 1 µg of RNA was used as a template. For each experiment, equal amounts of RNA were used for all samples as a template for cDNA manufacture. cDNA was made from template RNA using a commercial kit (Quantitect Reverse Transcriptase kit, Quiagen Ltd. Crawley UK). cDNA was stored at −20°C until use. Real-time quantitative RT-PCR was performed using a commercial thermal-cycler (Applied Biosciences 7900HT) with its associated software (Applied Biosciences SDS version 2.2.2). Real-time PCR reagents were all obtained commercially (Roche Diagnostics Ltd., Burgess Hill, UK). The 5′ nuclease technique based on Taq-polymerase and FAM labelled hydrolysis probes was used for all real-time reactions using commercially designed primer/probe combinations (Roche Universal Probe Library). Sequences and accession numbers used for primers and probes detecting *mHIF-1alpha*, *mHIF-2alpha* and *mβ-actin* are shown in Table 2. All reactions were performed in duplicate or triplicate. For all test samples, the endogenous control β-actin was used to verify equal loading and to facilitate relative quantitation calculation. Endogenous control samples were loaded on the same plate as the test samples, from the same aliquot of cDNA. A water-only control sample was included for each primer/probe combination on each test plate to verify the lack of contaminants in the mixture. For each experiment, a suitable control sample was used to compare the test samples. Amplicon-specific standard curves were produced using mHIF-1alpha and mHIF-2alpha DNA of known concentration. Ct values of the samples were compared to the standard curves and resultant cDNA concentrations were expressed in pg/µl. The -ΔCt method of relative quantitation was used to compare data, and only data from samples in a single plate run at the same time were compared statistically.

**Table 2:** Primers and probes used for real-time RT-PCR.

Primer/probe name	Gene accession number (NCBI)	Sequence
*mHIF-1alpha* forward	NM_010431	catgatggctccctttttca
*mHIF-1alpha* reverse	NM_010431	gtcacctggttgctgcaata
*mHIF-1alpha* probe	NM_010431	cagcagga
*mHIF-2alpha* forward	NM_010137	gggaacactacacccagtgc
*mHIF-2alpha* reverse	NM_010137	tcttcaagggattctccaagg
*mHIF-2alpha* probe	NM_010137	cagcagcc
*mβ-actin* forward	NM_007393	aaggccaaccgtgaaaagat
*mβ-actin* reverse	NM_007393	gtggtacgaccagaggcatac
*mβ-actin* probe	NM_007393	tgctgtcc

### Western blotting

Retinal tissue (pooled from 4 eyes for each time-point) was lysed using a commercial buffer (RIPA, Sigma Aldrich, Gillingham, UK) with added protease inhibitor cocktail (Sigma Aldrich, Gillingham, UK). Cell membranes were disrupted using a sonicator with a micro-tip (Soniprep 150, MSE London UK). Lysates were archived at −80°C. Equal amounts of protein were run on a 7.5% (w/v) reducing SDS polyacrylamide electrophoresis gel. After transfer to PVDF membrane (Millipore Watford UK) and blocking in 5% (w/v) non fat milk, 1% (w/v) bovine serum albumin in PBST, membranes were incubated with primary antibodies as detailed in Table 1. After washing in PBST, secondary antibody was incubated at a concentration of 1∶5000 - 1∶10 000 for 1 hour at room temperature (Pierce Immunopure goat anti-rabbit and anti-mouse IgG (H+L) HRP conjugated, Perbio Science UK Ltd., Northumberland UK). Chemiluminescence detection was performed using a Fujifilm LAS-1000 Luminescence Image Analyser after incubation with enhanced luminescence reagent (ECL plus GE Healthcare UK Ltd. Amersham, UK). Western blots were all performed in duplicate.

### ELISA for mVEGFA and mEpo

Whole eyes were collected from mice and snap frozen in liquid nitrogen. Eyes were homogenised in sterile PBS with protease inhibitors (Sigma Aldrich, Gillingham UK) using a glass homogeniser. The homogenate was spun at 7000rpm for 10 minutes. Protein concentration of the supernatant was measured using a Lowry-based colorimetric protein assay performed in triplicate (DC protein assay kit, Bio-Rad, Hemel Hempstead UK) compared to a bovine serum albumin standard curve. Sample endpoint ELISA was measured in triplicate using a microplate reader (Emax, Molecular Devices Ltd. Berkshire UK) comparing the optical density at 450 nm with a reference at 650 nm. The combined quantity of murine VEGF-A 164 and 120 splice variants (Mouse VEGF DuoSet ELISA development kit, R and D Systems Europe Abingdon UK) or murine erythropoietin (Mouse erythropoietin Quantikine ELISA development kit, R and D Systems) was calculated per milligram of whole eye protein comparing each sample to a standard curve of known concentration.
